# Affective and contextual values modulate spatial frequency use in object recognition

**DOI:** 10.3389/fpsyg.2014.00512

**Published:** 2014-05-28

**Authors:** Laurent Caplette, Gregory West, Marie Gomot, Frédéric Gosselin, Bruno Wicker

**Affiliations:** ^1^Département de Psychologie, CERNEC, Université de MontréalMontréal, QC, Canada; ^2^INSERM U930 Imagerie et Cerveau, Université François-Rabelais de ToursCHRU de Tours, Tours, France; ^3^CNRS UMR 7289, Institut de Neurosciences de la Timone, Aix-Marseille UniversitéMarseille, France

**Keywords:** object recognition, internal representations, affective value, context, spatial frequencies

## Abstract

Visual object recognition is of fundamental importance in our everyday interaction with the environment. Recent models of visual perception emphasize the role of top-down predictions facilitating object recognition via initial guesses that limit the number of object representations that need to be considered. Several results suggest that this rapid and efficient object processing relies on the early extraction and processing of low spatial frequencies (LSF). The present study aimed to investigate the SF content of visual object representations and its modulation by contextual and affective values of the perceived object during a picture-name verification task. Stimuli consisted of pictures of objects equalized in SF content and categorized as having low or high affective and contextual values. To access the SF content of stored visual representations of objects, SFs of each image were then randomly sampled on a trial-by-trial basis. Results reveal that intermediate SFs between 14 and 24 cycles per object (2.3–4 cycles per degree) are correlated with fast and accurate identification for all categories of objects. Moreover, there was a significant interaction between affective and contextual values over the SFs correlating with fast recognition. These results suggest that affective and contextual values of a visual object modulate the SF content of its internal representation, thus highlighting the flexibility of the visual recognition system.

## Introduction

Rapid and accurate visual recognition of everyday objects encountered in different orientations, seen under various illumination conditions, and partially occluded by other objects in a visually cluttered environment is necessary for our survival. The first theoretical efforts to explain this feat relied on purely bottom-up mechanisms in the visual system: cells in early visual areas would be sensitive to low-level features and cells in higher areas would integrate this information in order to then match it to a representation in memory (e.g., Maunsell and Newsome, 1987). However, it is improbable that feedforward pathways alone can account for object recognition because of their severely limited information processing capabilities (Gilbert and Sigman, 2007). Moreover, since these early theoretical efforts, the essential role of such feedback mechanisms in vision has been amply demonstrated (e.g., Rao and Ballard, 1999; Tomita et al., 1999; Barceló et al., 2000; Pascual-Leone and Walsh, 2001). Nowadays, most top-down models of object recognition (e.g., Grossberg, 1980; Ullman, 1995; Friston, 2003) propose that the search for correspondence between the input pattern and the stored representations is a bidirectional process where the input activates bottom-up as well as top-down streams that simultaneously explore many alternatives; object recognition is achieved when the counter streams meet and a match is found. The content of these stored representations could depend on several factors such as task requirements (e.g., perception or action, basic-level vs. superordinate-level categorization) or categorical properties of the object (e.g., animate vs. inanimate, affective vs. non-affective, social vs. non-social; Logothetis and Sheinberg, 1996). Understanding the properties of the stored representations that lead to the generation of predictions thus is an important unexplored issue. In particular, it remains to be understood if different representational systems are used during recognition of different categories of visual objects.

Building on the predictive account of visual object recognition, Bar (2003) proposed a brain mechanism for the cortical activation of top-down processing during object recognition, where low spatial frequencies (LSFs) of the image input are projected rapidly and directly through quick feedforward connections, from early visual areas into the dorsal visual stream. Such LSF information activates a relatively small set of probable candidate interpretations of the visual input in higher prefrontal integrative centers. These initial guesses are then back-projected along the reverse hierarchy to guide further processing and gradually encompass high spatial frequencies (HSFs) available at lower cortical visual areas. This proposal is supported by neurophysiological, computational and psychophysical evidence that LSFs are processed earlier than HSFs (Watt, 1987; Schyns and Oliva, 1994; Bredfeldt and Ringach, 2002; Mermillod et al., 2005; Musel et al., 2012; for reviews, see Bullier, 2001; Bar, 2003; Hegdé, 2008) and that top-down processing in visual recognition relies on LSFs (Bar et al., 2006); moreover, magnocellular projections, which are more sensitive to LSFs (Derrington and Lennie, 1984), seem to be implicated in initiation of top-down processing (Kveraga et al., 2007). Stored internal representations may thus be biased toward LSFs, since objects would be primarily matched in memory with an LSF draft.

Only a handful of studies have focused on the effect of specific SF band filtering during object recognition. In a name-picture verification task, low-pass filtering selectively impaired subordinate-level category verification (e.g., verify the “Siamese” category instead of the “animal” category at the superordinate level or the “cat” category at the basic level), while having little to no effect on basic-level category verification, suggesting that basic-level categorization does not particularly rely on LSFs (Collin and McMullen, 2005). On the other hand, Harel and Bentin (2009) reported that subordinate-level categorization was impaired by the removal of HSFs, but also that basic-level categorization was equally impaired by removal of either HSFs or LSFs, thus suggesting that neither of these bands is especially useful for recognition at the basic level. Finally, using a superordinate-level categorization task, Calderone et al. (2013) reported no difference in accuracy or response times between LSFs and HSFs. Overall, these studies suggest that, although this seems a bit different for subordinate-level categorization, neither LSFs or HSFs have a privileged role in object recognition. Even if LSFs do initiate a top-down processing, this suggests that their overall role in recognition is negligible; other SFs (neither low or high), however, may have a preponderant role.

Intrinsic properties of visual objects such as their affective value or contextual associativity may modulate the content of internal representations. Because of their great adaptive value, emotional objects might necessitate fast recognition, to facilitate an immediate behavioral response; this is likely to apply to both dangerous and pleasant stimuli, the former threatening survival and the latter promoting it (Bradley, 2009). In fact, the brain's prediction about the identity of a visual object may be partly based on its affective value, i.e., prior experiences of how perception of a given object has influenced internal body sensations. As such, affective value could be not just a label or judgment applied to the object post-recognition, but rather an integral component of mental object representations (Lebrecht et al., 2012) and could act as an additional clue to the object's identity to facilitate its recognition (Barrett and Bar, 2009). Since emotional objects need to be processed quickly, it is likely that LSFs, which are extracted rapidly, are particularly important for their recognition. In agreement with this idea, there is some evidence that LSFs are more present in representations of objects with strong affective value than in representations of neutral objects. Mermillod et al. (2010) reported that threatening stimuli were recognized faster and more accurately than neutral ones with LSFs but not with HSFs. Other behavioral and neuroimaging studies also suggested an interaction between emotional content and LSFs in various perceptual tasks. Bocanegra and Zeelenberg (2009), for instance, observed that in a Gabor orientation discrimination task, briefly presented fearful faces improved subjects' performance with LSF gratings while impairing it with HSF gratings. Moreover, early ERP amplitudes sensitive to affective content were found to be greater when unpleasant scenes were presented intact or in LSFs rather than in HSFs (Alorda et al., 2007). In the same vein, Vuilleumier et al. (2003) observed that the amygdala responded to fearful faces only if LSFs were present in the stimulus. In an intracranial ERP study where subjects were presented with both visible and invisible (masked) faces, Willenbockel et al. (2012) found that amygdala activation correlated mostly with SFs around 2 and 6 cycles/face, while insula activation correlated mostly with slightly higher SFs near 9 cycles/face. All these results suggest that the internal representations of objects with affective value would comprise more LSFs than representations of neutral objects.

Relatedly, the contextual associativity of a visual object—“what other objects or context might go with this object?” (Bar, 2004; Fenske et al., 2006)—could also impact on the SF content of its mental representation. It has been shown that recognition of an object that is highly associated with a certain context facilitates the recognition of other objects that share the same context (e.g., Bar and Ullman, 1996). A lifetime of visual experience would lead to contextual associations that guide expectations and aid subsequent recognition of associated visual objects through rapid sensitization of their internal representations (Biederman, 1972, 1981; Palmer, 1975; Biederman et al., 1982; Bar and Ullman, 1996). This associative processing is quickly triggered merely by looking at an object and would be critical for visual recognition and prediction (Bar and Aminoff, 2003; Aminoff et al., 2007). It has been suggested that the rapidly extracted LSFs of an object image are sufficient to activate these associated representations, and thus that the representations of contextual objects are likely to be biased toward LSFs (Bar, 2004; Fenske et al., 2006). However, this hypothesis has never been tested directly.

Affective and contextual values may also interact, so that representations of visual objects with affective value could be modulated by their contextual value or vice-versa (e.g., Storbeck and Clore, 2005; Brunyé et al., 2013; Shenhav et al., 2013). Indeed, the affective value of a given object is often defined by the context to which it has been associated to in memory. For example, a tomb elicits sadness, not because it is inherently sad, but because it evokes a context of cemetery/death. As such, affective objects might be differentially represented whether or not their affective value originates from their associated contexts. Interactions between both psychological properties have been reported. For instance, our affective state influences the breadth of the associations we make (Storbeck and Clore, 2005) and conversely, the generation of associations influences our affective state (Brunyé et al., 2013). Also, it seems that associative and affective processing both take place in the medial orbitofrontal cortex, and that both contextual and affective values might in fact relate to a more unified purpose (Shenhav et al., 2013).

The current study examined the SF content of stored internal representations of visual objects with different affective and contextual values, by evaluating what are the SFs in the stimuli that correlate with fast and accurate identification. Stimuli consisted of pictures of objects equalized in SF content and categorized as having low or high affective and contextual values. The SFs of these stimuli were randomly sampled on a trial-by-trial basis while subjects categorized the objects portrayed in the images. By varying affective value, contextual value and spatial frequencies available in the object image altogether, we aimed to clarify their roles in visual recognition, and to study potential interactions between them.

## Methods

### Participants

Forty-seven healthy participants (33 males) with normal or corrected-to-normal visual acuity were recruited on the campus of the Université de Montréal for an object recognition study. Participants were aged between 19 and 31 years (*M* = 23.04; *SD* = 3.13) and did not suffer from any reading disability. A written informed consent was obtained prior to the experiment, and a monetary compensation was provided upon its completion.

### Apparatus

The experimental program was run on a Mac Pro computer in the Matlab (Mathworks Inc.) environment, using functions from the Psychophysics Toolbox (Brainard, 1997; Pelli, 1997). A refresh rate of 120 Hz and a resolution of 1920 × 1080 pixels were set on the Asus VG278H monitor used for stimuli presentation. The relationship between RGB values and luminance levels was linearized. Luminance depth was 8 bits, and minimum and maximum luminance values were 1.1 cd/m^2^ and 134.0 cd/m^2^, respectively. A chin rest was used to maintain viewing distance at 76 cm.

### Stimuli

#### Selection and validation

**Figure 1:**
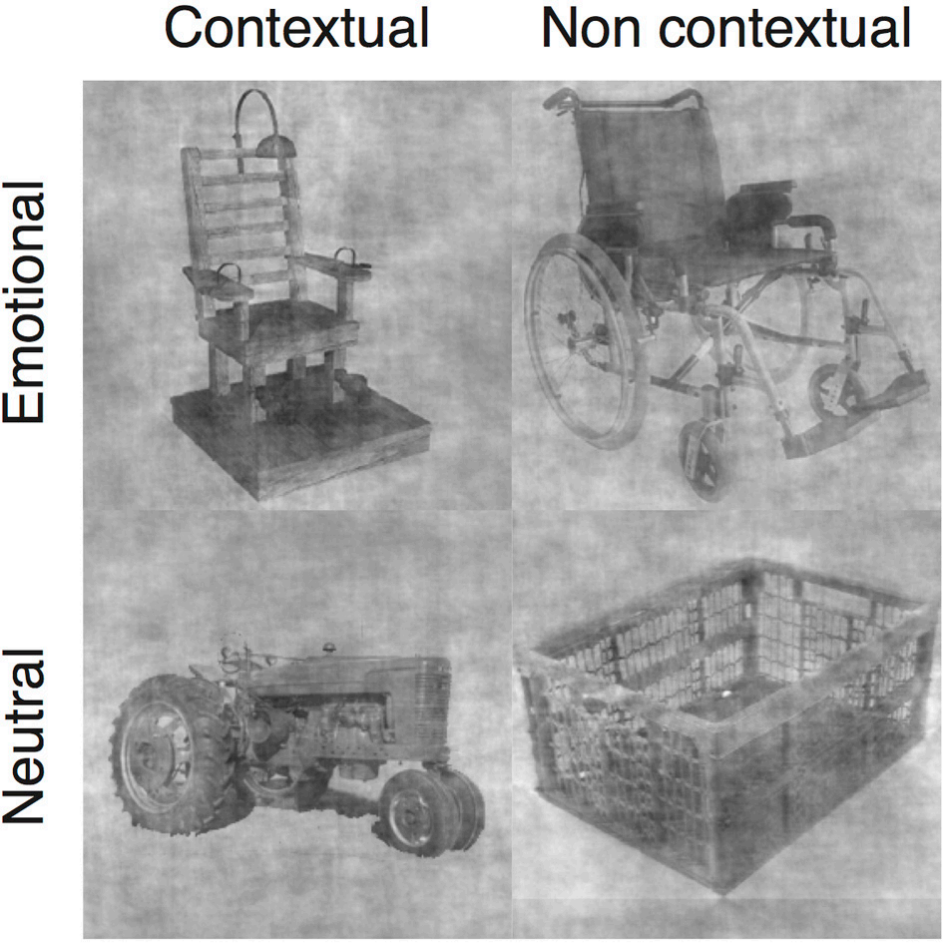
**Example images for each of the four categories of objects**.

One hundred fifty six object images were pre-selected mainly from the database used in Shenhav et al. (2013) but also from Internet searches. Each object image was presented to 30 raters who decided either (i) if they associated the object to a particular emotion, and if so, to which one or (ii) if they associated the object to a particular context, and if so, to which one. For the experiment, we selected 18 objects with clear consensus (or absence of) regarding their contextual and affective values in each of our four object categories: contextual emotional, non-contextual emotional, contextual neutral and non-contextual neutral (Figure 1, Table S1). Clear consensus about high affective or high contextual value meant that an object was associated to the same context or to the same emotion by more than 75% of raters; and clear consensus about low affective or contextual value meant that an object was associated to no particular context or emotion by more than 75% of the raters. Fifty-one of the selected images came from the Shenhav et al. (2013) database, and our affective and contextual ratings for these images closely matched theirs.

#### Control of low-level features

Stimuli thus consisted of 72 grayscaled object images of 256 × 256 pixels presented on a mid-gray background. The images subtended 6 × 6° of visual angle. Median object width was equal to 237 pixels. To target our investigation on stored internal representations and get rid of a potential interaction between the visual input and the representation, spatial frequency content and luminance were equalized across stimuli using the SHINE toolbox (Willenbockel et al., 2010a). Resulting images had a RMS contrast of 0.075. We reduced the undesired impact of psycho-linguistic factors, such as word length and lexical frequency, on response times by transforming these into z-scores for every object. For example, we computed the mean and standard deviation of the RTs of the correct positive trials in which the electric chair was presented, and we used these statistics to transform those RTs into z-scores. We did the same for all the other objects. As a result, the means and standard deviations of the RTs associated with every word were strictly identical, and all RT variations due to differences between the words were eliminated.

#### Sampling

**Figure 2:**
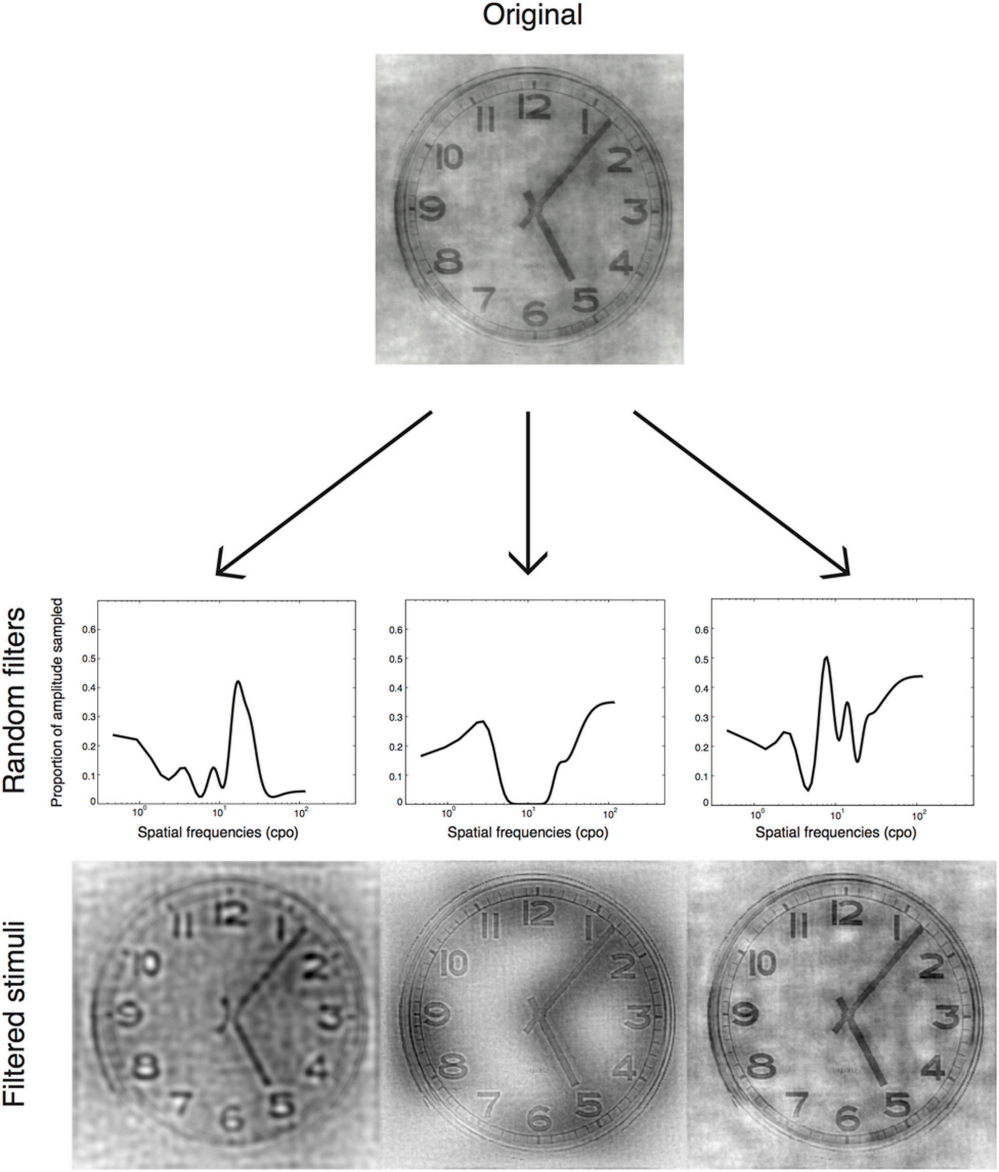
**Examples of stimuli presented in the experiment**. These are generated by applying random filters to a base image.

SF content of the images properly padded was extracted via Fast Fourier Transform (FFT) and randomly filtered at each trial, according to the SF Bubbles method (Willenbockel et al., 2010b). In short, each spatial frequency filter was created by first generating a random vector of 10,240 elements consisting of 20 ones (the number of bubbles) among zeros. Second, the resulting vector was convolved with a Gaussian kernel that had a standard deviation of 1.8. Third, the vector was log transformed so that the SF sampling approximately fit the SF sensitivity of the human visual system (see De Valois and De Valois, 1990). The resulting sampling vector contained 256 elements representing each spatial frequency from 0.5 to 128 cycles per image. To create the two-dimensional spatial frequency filtered images, vectors were rotated about their origins and dot-multiplied with the FFT amplitudes (see Willenbockel et al., 2010b, for methodological details). Thus, several SF bandwidths were revealed in each stimulus; and objects were presented several times with different SF bandwidths revealed every time (Figure 2).

### Procedure

After they had completed a short questionnaire for general information (age, sex, education, language, etc.), participants sat comfortably in front of a computer monitor, in a dim-lighted room. Participants did two 500-trial blocks, with a short break in between. Each trial began with a central fixation cross lasting 300 ms, followed by a blank screen for 100 ms, the SF-filtered random object image for 300 ms, a central fixation cross for 300 ms, a blank screen for 100 ms, and finally a matching or mismatching object name that remained on the screen until the participant had answered or for a maximum of 1000 ms. Subjects were asked to indicate with a keyboard key press as accurately and rapidly as possible whether or not the name matched the object depicted in the image. This picture-name verification task was chosen because it imposes a specific level of categorization to subjects (we chose the basic-level) without focusing attention explicitly on either affective or contextual value of the object. Name and object matched on half the trials.

### Spatial frequency data analysis

To determine the spatial frequencies that contributed most to fast object recognition for each condition, we performed least-square multiple linear regressions between RTs and corresponding sampling vectors. Only correct positive trials (i.e., when the name matched the object, and the participant answered correctly) were included in the analysis. RTs were first z-scored for every object to minimize undesired sources of variability pertaining to psycho-linguistic factors such as word length and lexical frequency (see Stimuli: Control of low-level features). They were further z-scored for each condition in each subject's session to diminish variability due to task learning. Trials associated with z-scores over 3 or below 3 were discarded (<1.8% of trials).

We call the resulting vectors of regression coefficients classification vectors. We first contrasted the classification vector for all objects against zero to examine what were the spatial frequencies used in general, regardless of affective or contextual values. We then contrasted the classification vectors for all emotional objects and all neutral objects, and the ones for all contextual objects and all non-contextual objects, to assess the main effects of contextual and affective values. Next, we examined if there was an interaction between these two dimensions. To do so, we contrasted classification vectors of all four subcategories of objects by applying the following formula:

$$\left(A_{1}B_{1}-A_{1}B_{2}\right)-\left(A_{2}B_{1}-A_{2}B_{2}\right),$$

where A represents emotional value, B represents contextual value, and the number represents the level of the variable. We finally investigated the simple effects by comparing the conditions pairwise. The statistical significance of the resulting classification vectors was assessed by applying the Cluster test (Chauvin et al., 2005). Given an arbitrary z-score threshold, this test gives a cluster size above which the specified *p*-value is satisfied. We used this test rather than the Pixel test (Chauvin et al., 2005) because it is in general more sensitive, allowing us to detect weaker but more diffuse signals. Here, we used a threshold of ±3 (*p* < 0.05, two-tailed). We report the size *k* of the significant cluster and its maximum Z-score *Z*_max_. We implemented the Cluster tests as bootstraps (Efron and Tibshirani, 1993); that is, we repeated all regressions 10,000 times pairing the sampling vectors with transformed RTs randomly selected in the observed transformed RT distribution. This resulted in 10,000 random classification vectors per condition. We used these random classification vectors to transform the elements of the observed classification vectors into z-scores and estimate their *p*-values. We corrected p-values for multiple comparisons in the pairwise comparisons by implementing Hochberg's step-up procedure (Hochberg, 1988).

## Results

### Effects of condition and spatial frequencies on accuracy

The mean accuracy was 87.49% (*SD* = 7.63). To analyse possible effects of condition on accuracy, without taking SFs into account, we first conducted a 2 (Context: non-contextual or contextual) × 2 (Emotion: neutral or emotional) repeated-measures ANOVA on mean accuracies per participant. There was an effect of contextual value [*F*_(1, 46)_ = 39.83, *p* < 0.001, η^2^_*p*_ = 0.46]: non-contextual objects (*M* = 81.92%; *SD* = 9.21) were recognized more easily than contextual ones (*M* = 77.19%; *SD* = 10.96). There also was an effect of emotional value [*F*_(1, 46)_ = 6.31, *p* < 0.05, η^2^_*p*_ = 0.12]: neutral objects (*M* = 80.30%; *SD* = 9.48) were recognized slightly more easily than emotional objects (*M* = 78.81%; *SD* = 10.49).

There was an interaction between emotional and contextual values [*F*_(1, 46)_ = 53.04, *p* < 0.001, η^2^_*p*_ = 0.53]. This interaction was decomposed into simple effects. First, there was an effect of emotion on non-contextual objects [*F*_(1, 46)_ = 49.63, *p* < 0.001, η^2^_*p*_ = 0.52]. Non-contextual neutral objects (*M* = 85.58%; *SD* = 7.94) were recognized more easily than non-contextual emotional objects (*M* = 78.26%; *SD* = 11.49). Second, there was an effect of emotion on contextual objects as well [*F*_(1, 46)_ = 20.87, *p* < 0.001, η^2^_*p*_ = 0.31]. Contextual emotional objects (*M* = 79.36%; *SD* = 10.31) were recognized more easily than neutral contextual objects (*M* = 75.02%; *SD* = 12.45).

Accuracy did not correlate significantly with the presentation of any SF.

### Effect of condition on response times

The mean RT for correct positive trials was 623 ms (*SD* = 83). To analyse possible effects of condition on RTs, without taking SFs into account, we conducted a 2 (Context: non-contextual or contextual) × 2 (Emotion: neutral or emotional) repeated-measures ANOVA on − log(x + 1)-transformed RT means per participant (Ratcliff, 1993). Aberrant scores (over 2 s) were excluded from the analysis. There was an effect of contextual value on RTs [*F*_(1, 46)_ = 161.29, *p* < 0.001, η^2^_*p*_ = 0.78] whereby non-contextual objects (*Md*^1^ = 596 ms; *SD* = 60) were recognized faster than contextual ones (*Md* = 537 ms; *SD* = 67). There was no effect of emotional value [*F*_(1, 46)_ < 1].

There also was an interaction between emotional value and contextual value [*F*_(1, 46)_ = 18.46, *p* < 0.001, η^2^_*p*_ = 0.29]. This interaction was decomposed into simple effects. First, there was an effect of emotion on non-contextual objects [*F*_(1, 46)_ = 12.53, *p* < 0.001, η^2^_*p*_ = 0.21]. Non-contextual neutral objects (*Md* = 532 ms; *SD* = 57) were identified faster than non-contextual emotional objects (*Md* = 548 ms; *SD* = 68). There also was an effect of emotion on contextual objects [*F*_(1, 46)_ = 10.15, *p* < 0.01, η^2^_*p*_ = 0.18]. Contextual emotional objects (*Md* = 579 ms; *SD* = 64) were identified faster than contextual neutral ones (*Md* = 609 ms; *SD* = 80).

### Effect of spatial frequencies on response time

**Figure 3:**
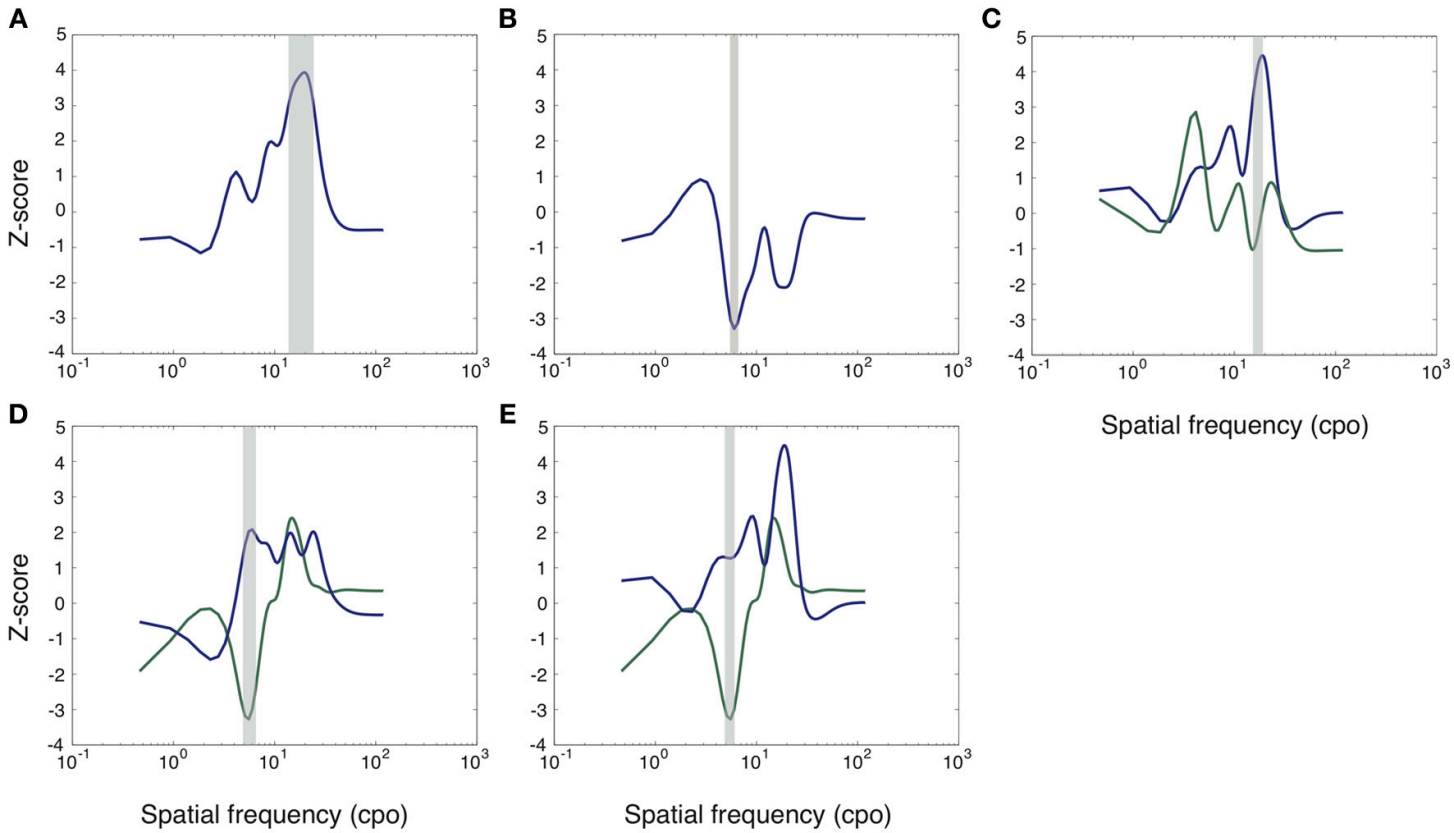
**Group classification vectors depicting the correlations between SFs and RTs for different conditions**. Higher z-scores indicate a negative correlation (SFs leading to shorter RTs) while lower z-scores indicate a positive correlation (SFs leading to longer RTs). Highlighted gray areas are significant (*p* < 0.05). See text for details. **(A)** All objects together. **(B)** The vector depicting potential interactions between both variables, obtained by contrasting the contrasts of contextual value for both levels of emotional value. **(C)** Non-contextual neutral (green) objects and contextual neutral (blue) objects. **(D)** Contextual emotional (green) and non-contextual emotional (blue) objects. **(E)** Contextual emotional (green) and contextual neutral (blue) objects.

To determine the spatial frequencies that contributed most to fast object recognition for each condition, we performed least-square multiple linear regressions between z-scored transformed RTs (see Methods: Spatial Frequency Data Analysis) and corresponding sampling vectors for correct positive trials. All object categories confounded, SFs between 13.71 and 24.31 cycles per object width (cpo) correlated negatively with RTs (peak at 19.45 cpo, *Z*_max_ = 3.94, *k* = 23, *p* < 0.01; Figure 3A). In other words, RTs were consistently reduced with the presentation of SFs within these boundaries. To examine a possible effect of emotional value, we contrasted classification vectors for all emotional objects and all neutral objects. There was no significant difference (*p* > 0.05). Similarly, there was no significant difference between non-contextual and contextual objects (*p* > 0.05).

We then examined the interaction between affective and contextual values (see Methods: Spatial frequency data analysis). We found a significant interaction for SFs between 5.52 and 6.69 cpo (peak at 6.02 cpo, *Z*_max_ = 3.29, *k* = 3, *p* < 0.05; Figure 3B).

We subsequently decomposed the interaction into simple effects. There was a significant effect of contextual value on neutral objects between 15.25 and 19.20 cpo; these SFs were correlated more negatively with RTs for contextual neutral objects than for non-contextual neutral objects (peak at 18.98 cpo, *Z*_max_ = 3.36, *k* = 9, *p* < 0.05, corrected for multiple comparisons; Figure 3C). However, the interaction was not significant for these SFs, making this effect difficult to interpret. There also was an effect of contextual value for emotional objects: SFs between 4.86 and 6.56 cpo correlated more positively with RTs for contextual emotional objects than for non-contextual emotional objects (peak at 5.56 cpo, *Z*_max_ = 3.75, *k* = 4, *p* < 0.05, corrected for multiple comparisons; Figure 3D). Moreover, there was an effect of emotional value on contextual objects: SFs between 4.86 and 6.09 cpo correlated more positively with RTs for contextual emotional objects than for contextual neutral objects (peak at 5.56 cpo, *Z*_max_ = 3.21, *k* = 3, *p* < 0.05, corrected for multiple comparisons; Figure 3E). Finally, we observed no significant difference between non-contextual neutral and non-contextual emotional objects (*p* > 0.05). The interaction thus seems to be caused by the significant effect of contextual value on emotional but not on neutral objects, combined with the significant effect of emotional value on contextual but not on non-contextual objects.

## Discussion

### General spatial frequency use

A few studies have examined the effect of specific SF band filtering during name-picture verification tasks, similar to ours. Collin and McMullen (2005) reported that low-pass filtering objects had little impact on basic-level verification (e.g., verify the “cat” category instead of the “animal” category at the superordinate level or the “Siamese” category at the subordinate level), suggesting that basic-level categorization does not especially rely on LSFs. Furthermore, Harel and Bentin (2009) reported that basic-level categorization was equally impaired by removal of either HSFs or LSFs, thus suggesting that neither of these bands is especially useful for recognition at the basic level. However, Harel and Bentin's cutoff for HSFs was especially high (65 cpo, or 6.5 cpd), thus preserving only very fine information typically not useful for object recognition. A large band of intermediate spatial frequencies was not explored in these studies.

An important aspect of our study is that instead of applying filters with fixed arbitrary cut-offs, we randomly sampled the entire SF spectrum. This allowed us to overcome the need of selecting arbitrary SF bands to evaluate. Indeed, there is no consensus in the literature about what consists of LSFs or HSFs: this seems to be more understood as a relative measure for SF bands inside a given study. Cut-offs for LSFs in the literature vary from 5 cpo (Boutet et al., 2003) to 15 cpo (Alorda et al., 2007). Similarly, cut-offs for HSFs vary from 20 cpo (Boutet et al., 2003) to 65 cpo (Harel and Bentin, 2009). When cut-offs are translated into cycles per degree (cpd), acknowledging that the diagnostic SFs may vary according to viewing distance, the discrepancy is even larger: cut-offs for LSFs vary from less than 0.4 cpd (Boutet et al., 2003) to more than 2.4 cpd (Alorda et al., 2007) and cut-offs for HSFs vary from 1.4 cpd (Boutet et al., 2003) to 6.5 cpd (Harel and Bentin, 2009). Quite interestingly, we note that some SFs (between 1.4 and 2.4 cpd) may be included either in LSFs or HSFs.

Our random sampling of the entire SF spectrum allowed us to evaluate the use of SFs considered as neither low nor high by most previous studies. Using this unbiased experimental approach, we found that intermediate SFs between about 14 and 24 cpo (2.3–4 cpd) are associated with fast RTs for basic-level verification. This suggests that objects are processed particularly rapidly through these SFs. Although this interpretation is the most straightforward, it is also possible that object processing was at least partly completed before the presentation of the words and, therefore, that the RTs reflect remnants of object processing rather than object processing *per se*.

Another unique aspect of our study is the fact that we equalized SF content of the object images prior to their sampling. This allows us to interpret results more confidently in terms of content of internal representations. Indeed, if SF content is not normalized among stimuli, results most likely reflect an interaction of the stored representation with the information available in the stimulus. Unfortunately, few studies have applied this procedure. As a notable exception, Willenbockel et al. (2010b) did equalize SF spectrum and randomly sample SFs in a face recognition task. Results revealed that SFs peaking at approximately 9 and 13 cycles/face (equivalent to 1.4 and 2 cpd, i.e., SFs that may be categorized as LSFs, HSFs, or most often neither of these) were most correlated with fast and accurate face identification. Although these SFs specific for images of faces are likely to differ from the SF content of object representations, they are an additional indicator that, as in the present study, intermediate SFs rather than LSFs occupy the greatest place in our representation of the world. It is plausible that stored representations consist of mostly these SFs because they are part of the intermediate band of SFs to which we are naturally most sensitive (e.g., Watson and Ahumada, 2005).

### Interaction between affective and contextual values

No main effect of contextual or affective value was observed in the SFs correlating with the objects' fast identification. However, we found a significant interaction between affective and contextual values for SFs centered on 6 cpo (or 1 cpd). This indicates that these LSFs, those usually associated with the magnocellular pathway (Derrington and Lennie, 1984), are sensitive in a non-linear manner to a combination of the visual object's intrinsic properties.

When testing the simple effects, we observed that affective value elicited a significant difference in the use of these SFs in contextual objects: they led to longer RTs for contextual emotional objects than for contextual neutral ones. This is not in accordance with the general effect of affective value usually reported in the literature (i.e., LSFs leading to faster RTs, e.g., Mermillod et al., 2010); however, our result is due to an interaction between affective and contextual values and is therefore difficult to compare to those of other studies. Moreover, our stimuli were equalized in their SF content and always comprised several randomly sampled SF bandwidths at the same time, whereas in studies using filters with fixed cut-offs, only some specific band of LSFs or HSFs is shown at a time.

SFs near 6 cpo (or 1 cpd) also led to longer RTs for contextual emotional objects than for non-contextual emotional objects. The effect of contextual value on SF content of object representations had not been tested before but it had been often proposed that rapidly extracted LSFs are sufficient to activate representations associated with an object (Bar, 2004; Fenske et al., 2006). Our data suggest that these presumed/hypothetical associative representations do not speed up the object's recognition. Why we observed this modulation only for emotional objects is not clear, but several interactions between affective and contextual processing have already been reported and could possibly explain the discrepancy (Storbeck and Clore, 2005; Brunyé et al., 2013; Shenhav et al., 2013). For example, affective value might influence the extent to which we associate a particular object to other objects (Bar, 2009; Shenhav et al., 2013).

## Conclusion

The main findings of the present study are (i) that the SF content of object representations in general are in an intermediate band between 14 and 24 cpo (2.3–4 cpd), and (ii) that intrinsic high-level categorical properties of an object influence the SF content of its internally stored representation, more precisely that affective and contextual values interact in their modulation of the SF content of object representations.

According to predictive accounts of brain function (e.g., Rao and Ballard, 1999; Bar, 2003; Friston, 2003, 2010; Friston et al., 2006), our mind constantly generates predictions about our environment, and our understanding of a sensory input is based both on the available sensory information and on prior beliefs stored as internal representations (see Knill and Pouget, 2004). In this study, we investigated precisely the SF content of these stored representations, and its potential flexible modulation by affective and contextual properties of the stimulus. Our results reveal that stored representations of visual objects are composed of intermediate SFs that are often left over in studies using filters with fixed arbitrary cut-offs. Furthermore, we observed a modulation of this SF content by affective and contextual intrinsic values of the visual object, suggesting its flexibility and thus the multiplicity of visual recognition systems.

Our study cannot however address directly the issue of temporal dynamics of visual object recognition. While we observed that some SFs are more useful to identify some objects, we cannot conclude that these are extracted first. Further studies should address these issues and their links to potential initiation of top-down mechanisms.

### Conflict of interest statement

The authors declare that the research was conducted in the absence of any commercial or financial relationships that could be construed as a potential conflict of interest.

## Footnotes

^1^Median reaction times are given, since the ANOVA was performed on log transformed values. Given that the mean log values wouldn't be readily interpretable and that the median values don't change with a log transformation, we made this choice for purposes of clarity and transparency.
