# Supplementary material for: Affective and contextual values modulate spatial frequency use in object recognition
Source: Front Psychol. 2014 May 28;5:512. doi: 10.3389/fpsyg.2014.00512 (PMC4036062; doi:10.3389/fpsyg.2014.00512)
Supplement: Supplementary file 1 [file DataSheet1.DOCX]

***Supplementary Material***

**Affective and contextual values modulate spatial frequency use in object recognition**

Laurent Caplette^1^, Greg L. West^1^, Marie Gomot^2^, Frédéric Gosselin^1^ & Bruno Wicker^3^

^1^CERNEC, Département de psychologie, Université de Montréal

^2^UMR-S ‘Imaging and Brain’, INSERM U930, CNRS ERL3106, Université François-Rabelais de Tours

^3^Institut de Neurosciences de la Timone, CNRS UMR 7289, Aix-Marseille Université

**Appendix A**

*List of the stimuli, along with the object category in which they were classified following our validation. The object names are the ones that were presented on the screen. We also provide an English translation.*

| **Object Category** | **Object Name** | **English Translation** |
| --- | --- | --- |
|  |  |  |
| *Contextual Emotional* | Berceau | Cradle |
|  | Bonhomme de neige | Snowman |
|  | Boule disco | Disco Ball |
|  | Brancard | Stretcher |
|  | Cadeau | Gift |
|  | Casque de soldat | Soldier's Helmet |
|  | Chaise longue | Deck Chair |
|  | Chaise électrique | Electric Chair |
|  | Fauteuil de dentiste | Dentist's Chair |
|  | Gâteau d'anniversaire | Birthday Cake |
|  | Hamac | Hammock |
|  | Masque et tuba | Snorkeling Mask |
|  | Mitraillette | Machine Gun |
|  | Palmes | Diving Fins |
|  | Pistolet | Handgun |
|  | Seau de plage | Beach Pail |
|  | Table de billard | Pool Table |
|  | Tombe | Tombstone |
| *Non-contextual Emotional* | Poubelle | Trash Can |
|  | Verre à cocktail | Cocktail Glass |
|  | Araignée | Spider |
|  | Billet de banque | Dollar Bill |
|  | Bombe | Bomb |
|  | Bonbon | Candy |
|  | Bouquet de fleurs | Flower Bouquet |
|  | Cadre pour photo | Picture Frame |
|  | Carré d'as | Four of a Kind |
|  | Crâne | Skull |
|  | Dynamite | Dynamite |
|  | Fauteuil roulant | Wheelchair |
|  | Grenade | Grenade |
|  | Téléphone cassé | Broken Phone |
|  | Papillon | Butterfly |
|  | Scie mécanique | Chainsaw |
|  | Tirelire | Piggy Bank |
|  | Verre de bière | Glass of Beer |
| *Contextual Neutral* | Baignoire | Bathtub |
|  | Casque de sécurité | Safety Helmet |
|  | Chariot d'épicerie | Grocery Cart |
|  | Cuisinière | Oven |
|  | Feu de circulation | Traffic Light |
|  | Gouvernail | Rudder |
|  | Marteau de tribunal | Gavel |
|  | Pompe à essence | Fuel Pump |
|  | Roulette de casino | Roulette |
|  | Réfrigérateur | Refrigerator |
|  | Satellite | Satellite |
|  | Sièges d'avion | Aircraft Seats |
|  | Sombrero | Sombrero |
|  | Tampon et encrier | Stamp and Ink |
|  | Tente | Tent |
|  | Tracteur | Tractor |
|  | Ventilateur | Fan |
|  | Voiturette de golf | Golf Cart |
| *Non-contextual Neutral* | Appareil photo | Camera |
|  | Banc | Bench |
|  | Bouteille | Bottle |
|  | Caisse en plastique | Plastic Crate |
|  | Chaussure | Shoe |
|  | Coussin | Cushion |
|  | Horloge | Clock |
|  | Jumelles | Binoculars |
|  | Lampe de poche | Flashlight |
|  | Livre | Book |
|  | Mouchoirs | Tissues |
|  | Panier | Basket |
|  | Pelote de ficelle | Ball of String |
|  | Prise électrique | Electrical Outlet |
|  | T-shirt | T-Shirt |
|  | Téléphone portable | Mobile Phone |
|  | Téléphone | Phone |
|  | Étagère | Shelf |
